# Impact of a diagnosis of ‘low-grade dysplasia’ in patients with Barrett’s esophagus

**DOI:** 10.1093/dote/doag028

**Published:** 2026-04-09

**Authors:** Chih-Han Kung, Ravi Vissapragada, Ann Schloithe, Tim Bright, Norma Bulamu, David Watson

**Affiliations:** Department of Surgery, Flinders Medical Centre, Bedford Park, Adelaide, SA, Australia; Department of Surgical and Perioperative Sciences, Surgery, Umeå University, Umeå, Sweden; Flinders Health and Medical Research Institute, College of Medicine and Public Health, Flinders University, Adelaide, SA, Australia; Department of Surgery, Flinders Medical Centre, Bedford Park, Adelaide, SA, Australia; Flinders Health and Medical Research Institute, College of Medicine and Public Health, Flinders University, Adelaide, SA, Australia; Department of Surgery, Flinders Medical Centre, Bedford Park, Adelaide, SA, Australia; Flinders Health and Medical Research Institute, College of Medicine and Public Health, Flinders University, Adelaide, SA, Australia; Flinders Health and Medical Research Institute, College of Medicine and Public Health, Flinders University, Adelaide, SA, Australia; Department of Surgery, Flinders Medical Centre, Bedford Park, Adelaide, SA, Australia; Flinders Health and Medical Research Institute, College of Medicine and Public Health, Flinders University, Adelaide, SA, Australia

**Keywords:** Barrett’s esophagus, low-grade dysplasia, radiofrequency ablation

## Abstract

Barrett’s esophagus (BE) is the precursor to esophageal adenocarcinoma (EAC). Progression to high-grade dysplasia (HGD)/EAC can be directly from non-dysplastic BE or via low-grade dysplasia (LGD). There is a lack of consensus about the implications of LGD diagnosis on progression risk and whether modifying risk progression with endoscopic interventions such as radiofrequency ablation (RFA) is appropriate. The aim of this study was to determine the clinical implications of LGD and the cost-effectiveness of different management strategies. Outcomes from a large single-center prospective BE surveillance database were retrospectively analyzed. All patients with BE in a structured surveillance program were included. The cohort was divided into three groups: non-dysplastic BE throughout, LGD at surveillance entry, and LGD developing during surveillance. Each group’s annual incidence of progression to HGD/EAC was calculated per 100 person-years. Outcomes were then applied within a health economic model for health economic analysis of ongoing endoscopic surveillance versus RFA of LGD to identify the most cost-effective management strategy. Nine hundred fourteen patients were included; 727 had non-dysplastic BE, 97 had LGD at entry, and 90 developed LGD during surveillance. Total surveillance time was 5212 person-years. Forty-six (5.0%) patients progressed to HGD/EAC, at an annual progression rate of 0.9 per 100 person-years. For subgroups, the progression rates to HGD/EAC were 0.6 per 100 person-years for non-dysplastic BE, 1.0 for LGD at entry, and 2.2 for LGD during surveillance (*P* < 0.0001). The most cost-effective management strategy was RFA if LGD was identified under surveillance, with an estimated cost per quality-adjusted life year gained being AU$26,763. Overall progression from LGD to HGD/EAC was comparable to most previous studies. RFA is a cost-effective management strategy for BE once LGD arises during surveillance.

## INTRODUCTION

Barrett’s esophagus (BE) is the recognized precursor for esophageal adenocarcinoma (EAC).^1^ Its reported prevalence varies widely with a lack of studies in unselected populations. In Australia, the prevalence has been reported to vary from 0.29% to 18.4% in selected symptomatic populations.^2^^,^^3^ A recent simulation study derived a plausible prevalence of 5.4% from known rates of EAC.^4^ Endoscopy surveillance for BE is the current standard of care, but it is accepted that histopathologic identification of higher risk patients, i.e. with low-grade dysplasia (LGD) or high-grade dysplasia (HGD), is subject to large interobserver variation,^5^^,^^6^ and it is generally recommended that the diagnosis of both LGD and HGD is confirmed by two expert pathologists.^7^

The impact of an LGD diagnosis, its risk of progression to HGD, and proposed management are still debated.^8–10^ Published studies show that annual progression rates from LGD to HGD vary widely from around 0.4 to 13.4 per 100 person-years,^11^ and a systematic review suggested a pooled progression rate of 1.73 per 100 person-years.^12^ In contrast, a study from the Netherlands stands out as an outlier, reporting a progression rate of 13.4 per 100 person-years.^13^ This is most likely due to different patient selection and thorough triple histopathological confirmation of LGD diagnoses within the context of a clinical trial protocol.

There is currently no uniformly agreed course of action once LGD is identified. Treatment recommendations include more frequent endoscopy surveillance versus endoscopic ablation, with radiofrequency ablation (RFA) now a commonly recommended approach.^8–10^ Studies have shown that ablation to completely eradicate the BE segment reduces the risk of progression, and complication rates from this are generally low.^13–15^ There have also been health economic studies in the USA that show the cost-effectiveness of RFA for LGD compared to enhanced surveillance.^16–18^ However, as both management options are recommended, and cost-effectiveness thresholds vary between health systems, further studies to assess the impact of an LGD diagnosis are warranted.

In the Southern Adelaide Local Health Network in Adelaide, South Australia, a structured, managed BE surveillance program has been in place since 2002.^19^ Patients diagnosed with BE at endoscopy are enrolled and endoscopy surveillance is managed according to an agreed evidence-based protocol. Experienced endoscopists carry out all surveillance endoscopies, and a program coordinator ensures adherence to correct timing for endoscopy and adequate biopsy sampling.^19^^,^^20^ The surveillance program has been continuously audited for over 20 years to ensure compliance with guidelines, and the database underpinning it now has more than 20 years of high-quality surveillance follow-up. Concurrently, we have developed and refined a health economic model for BE surveillance.^21^ In this study, we aimed to determine the clinical implications of a pathological diagnosis of LGD and assess the cost utility of different management strategies.

## METHODS

All patients managed in the Southern Adelaide Local Health Network in Adelaide, South Australia, with confirmed BE defined as visible metaplastic columnar mucosa in the esophagus at endoscopy and histopathology confirmation of intestinal metaplasia, are followed up through a structured endoscopy surveillance program. The program ensured appropriate scheduling of endoscopy and adherence to an appropriate biopsy technique according to the Seattle protocol. Data were collected prospectively and stored in a database that was used to coordinate surveillance recalls. This study retrospectively analyzed outcomes for patients undergoing endoscopy surveillance from January 2002 to February 2023. Individuals under surveillance were managed with high-dose proton pump inhibitor (PPI) therapy, and a dedicated coordinator ensured compliance with therapy, managed endoscopy surveillance recalls, and ensured adherence to the endoscopy surveillance biopsy protocol. The protocol, the structure of the program, and protocol adherence have been described previously.^19^^,^^20^

Identified patients were assigned to one of three groups: (1) patients with non-dysplastic BE who were never diagnosed with LGD; (2) patients with LGD at the first (index) endoscopy within the surveillance program; and (3) patients who initially had non-dysplastic BE but developed LGD during a subsequent surveillance endoscopy. Patients with HGD at entry were excluded, as well as patients who did not have at least one follow-up endoscopy procedure.

LGD was determined by histopathology assessment of endoscopic mucosal biopsies. Reflecting common practice in many parts of the world, the LGD diagnosis was not routinely reviewed by a separate independent expert pathologist. The index pathology was reported by a general pathologist in the community or a gastrointestinal pathologist, depending on where the initial endoscopy was performed. However, all subsequent pathology samples for patients in the managed surveillance program were reported by experienced gastrointestinal pathologists who were local experts in this field. During the study period, a finding of LGD was followed by an early follow-up endoscopy 6–12 months later. No patients with LGD underwent endoscopic ablation or any other endoscopic intervention during the study period. Any diagnosis of HGD or mucosal cancer was double-reported and reconfirmed at a second endoscopy. The primary outcome for this study was progression to either HGD or EAC.

Progression to HGD/EAC was determined and expressed as an annual rate per 100 person-years. Follow-up time was counted from the date of the index endoscopy to the date of the confirmed endoscopic finding of HGD/EAC or to the last recorded surveillance endoscopy in patients who had not progressed beyond LGD. The index endoscopy was defined as the first endoscopy in the non-dysplastic group and the LGD during the surveillance group, or the date of the endoscopy where LGD was diagnosed in the LGD at commencement of surveillance group. Cases of death prior to/without a diagnosis of HGD/EAC during follow-up were censored at the time of death.

Data were obtained from the surveillance database and presented as either actual numbers with percentages or as continuous data presented as mean ± standard deviation. Data were analyzed with the chi-square test, Fisher’s exact test, or analysis of variance (ANOVA). The risk of progression to HGD/EAC is presented as the annual rate per 100 person-years. Multivariable analysis of the risk of progression for the respective groups was carried out with a Cox proportional hazard model adjusting for gender, maximum length of BE (index M-length), smoking, and age. Significance was set as *P* < 0.05. The model was predetermined as it included the risk factors previously validated in the progression of Barrett’s (PIB) risk score^21^ with the addition of age. The Kaplan–Meier chart was used to visualize the risk of progression during follow-up with the log-rank test. Statistical analysis was performed using SPSS (version 25, IBM, Armonk, NY).

### Cost-effectiveness model

**Fig. 1:**
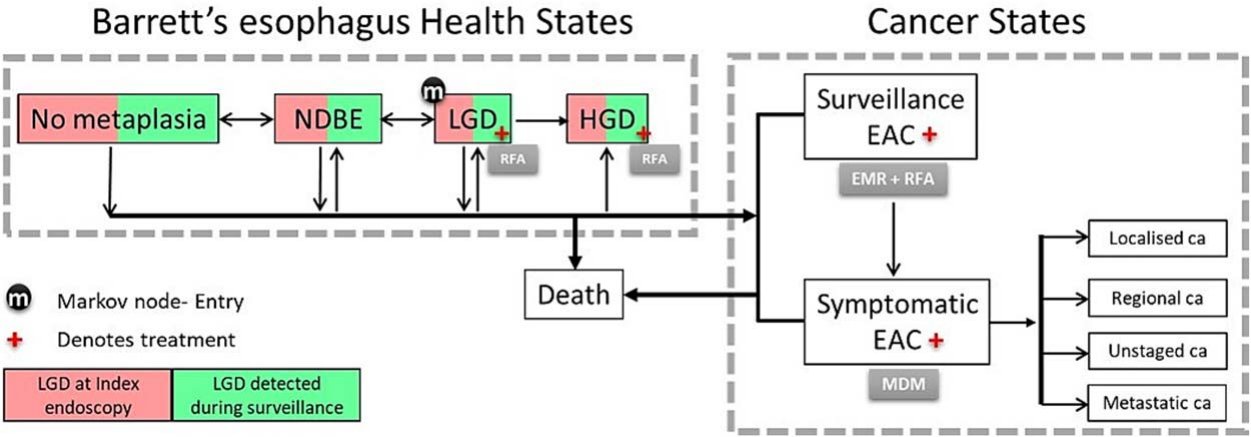
Markov model for health states and movements. EAC, esophageal adenocarcinoma; EMR, endoscopic mucosal resection; HGD, high-grade dysplasia; LGD, low-grade dysplasia; MDM, multidisciplinary meeting for stage-specific treatment; NDBE, non-dysplastic Barrett’s esophagus; RFA, radiofrequency ablation.

Cost-utility analysis was performed using a previously described Markov model of progression from BE to EAC.^22^ The original model was modified to include LGD as the starting population and incorporated progression rates calculated from the current study. Health states and the movements between states are depicted in Fig. 1. Cycle length for the model was 6 months, and the time horizon was 35 years with costs and outcomes discounted at 5%. The analysis was undertaken from an Australian health system perspective. The model outputs were expressed as cost in Australian dollars per quality-adjusted life year (QALY) gained, and incremental cost-effectiveness ratios below the Australian willingness-to-pay threshold of AU$50,000/QALY were considered cost-effective. The base case scenario modeled was a cohort of patients with LGD where ~52% commenced surveillance for the first time and ~48% progressed to LGD under surveillance. In the base case, all LGDs were subject to endoscopy surveillance every 6 months until HGD was detected, at which stage these individuals underwent endoscopic ablation with RFA. EAC detected through surveillance at an early stage (T1a) underwent endoscopic mucosal resection and then RFA ablation of any residual BE, whereas advanced EAC (>T1a) underwent stage-appropriate treatment including surgery, chemotherapy, and radiotherapy combinations. Three other strategies were included in the analysis: (1) RFA if LGD was detected during surveillance, (2) RFA if LGD was seen at the index endoscopy (after second endoscopy confirmation), and (3) RFA for all individuals with LGD.

**Fig. 2:**
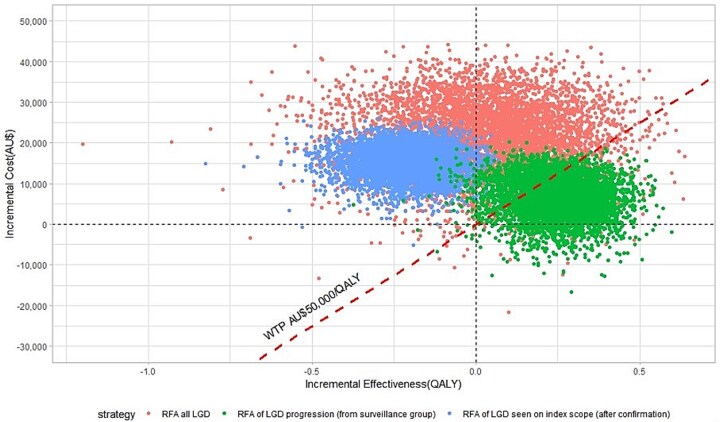
Probabilistic sensitivity analysis of 5000 simulations analyzing the cost-effectiveness of RFA.

Transition probabilities were derived using a seven-stage calibration process^23^^,^^24^ simulating the progression of the 187 patients with LGD in our database over 1341 person-years (Supplementary Table 1). Probabilities that could not be obtained from our data were derived from the literature (Supplementary Table 2). Costs were derived from a local South Australian database, and utility/disutility values from literature values were applied as either ‘per cycle’ or ‘per event’ (Supplementary Table 3). Deterministic (one-way sensitivity analysis) and probabilistic sensitivity analyses were performed to outline key drivers of the model and calculate error/confidence intervals, respectively. In one-way sensitivity analysis, inputs were varied one at a time across specific ranges to highlight the impact of the individual variable. These varied input values to see whether cost-effectiveness was dependent on one or more variables. In the probabilistic sensitivity analysis, 5000 simulations were run, where input values (transition probabilities, costs, utility/disutility values, etc.) with known values were varied according to specific distributions: gamma distributions for cost and beta distributions for probabilities and utility values (Fig. 2).

This study was approved by the Southern Adelaide Clinical Human Research Ethics Committee with reference number LNR/23/SAC/99 and conforms with the Helsinki Declaration.

## RESULTS

**Fig. 3:**
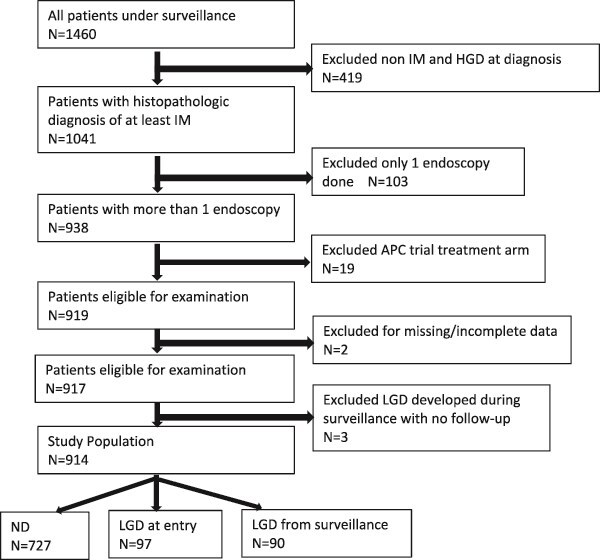
Flow chart for patient selection. APC, argon plasma coagulation; HGD, high-grade dysplasia; IM, intestinal metaplasia; LGD, low-grade dysplasia; ND, non-dysplastic.

Nine hundred and fourteen patients with BE were identified and met the inclusion criteria. Seven hundred and twenty-seven (79.5%) had non-dysplastic BE at the index and all follow-up endoscopies, 97 (10.6%) had LGD diagnosed at the index entry endoscopy, and 90 (9.8%) had non-dysplastic BE at the index endoscopy and then LGD identified at a subsequent surveillance endoscopy. Figure 3 summarizes the selection of patients for the study. The 914 patients had a mean age of 62.2 yrs and underwent a median of four surveillance endoscopies with a mean follow-up of 5.7 years. The total person-year follow-up for the cohort was 5212 years.

Baseline characteristics of the overall cohort and the study groups are summarized in Table 1. Endoscopy findings are summarized in Table 2. The median index M-length of the BE was longest in the group developing LGD during surveillance (5 cm) and shortest in the patients who never developed LGD (2 cm). For patients developing LGD during surveillance, the mean time from the index endoscopy to progression to LGD was 3.6 years. Variation in dysplasia diagnoses and the length of the BE segment is summarized in Table 3. Length variation and persistence or resolution of LGD were similar across all groups.

**Table 1:** Baseline characteristics of patients

	ND (*n* = 727)	LGD at entry (*n* = 97)	LGD during surveillance (*n* = 90)	All patients (*n* = 914)	*P*-value
Sex					0.603
Female	214 (29.4%)	29 (29.9%)	22 (24.4%)	265 (29.0%)	
Male	513 (70.6%)	68 (70.1%)	68 (75.6%)	649 (71.0%)	
Age (mean ± SD)	61.5 ± 14.3	65.6 ± 14.8	64.3 ± 13.9	62.2 ± 14.4	0.012
BMI (mean ± SD)	29.7 ± 6.1 (*n* = 374)	30.1 ± 4.6 (*n* = 42)	28.5 ± 5.2 (*n* = 52)	29.6 ± 5.9 (*n* = 468)	0.321
Number of endoscopies (median and range)	3 (2–14)	6 (2–26)	6 (2–17)	4 (2–26)	N/A
Follow-up time years (mean ± SD)	5.3 ± 4.4	7.2 ± 5.8	7.1 ± 5.0	5.7 ± 4.7	N/A
Total person-year follow-up	3872	699	642	5212^*^	N/A

BMI, body mass index; LGD, low-grade dysplasia; ND, non-dysplastic; SD, standard deviation; N/A, not available.

^*^Total numbers do not add up due to rounding. Analysis done with the chi-square test for categorical variables and ANOVA for continuous variables.

**Table 2:** Length of Barrett’s esophagus at endoscopy

	ND (*n* = 727)	LGD at entry (*n* = 97)	LGD during surveillance (*n* = 90)	All patients (*n* = 914)	*P*-value
Index C (median and range) (cm)	2 (0–20) (*n* = 664)	3 (0–16) (*n* = 92)	4 (0–15) (*n* = 88)	2 (0–20) (*n* = 844)	<0.01
Index M (median and range) (cm)	2 (0–20) (*n* = 664)	4 (0–17) (*n* = 92)	5 (1–15) (*n* = 88)	2 (0–20) (*n* = 844)	<0.01
Longest M (median and range) (cm)	3 (0–20) (*n* = 723)	4 (1–19) (*n* = 97)	7 (1–20) (*n* = 90)	3 (0–20) (*n* = 910)	<0.01
Length of BE at index endoscopy					<0.01
Missing	63 (8.7%)	5 (5.1%)	2 (2.2%)	70 (7.7%)	
≤2 cm	364 (50.1%)	34 (35.1%)	30 (33.3%)	428 (46.8%)	
>2–<5 cm	136 (18.7%)	21 (21.6%)	12 (13.3%)	169 (18.5%)	
5–<10 cm	125 (17.2%)	28 (28.9%)	33 (36.7%)	186 (20.4%)	
≥10 cm	39 (5.4%)	9 (9.3%)	13 (14.4%)	61 (6.7%)	
Longest length of BE at any endoscopy					<0.01
Missing	4 (0.6%)	0	0	4 (0.4%)	
≤2 cm	303 (41.7%)	26 (26.8%)	16 (17.8%)	345 (37.7%)	
>2–<5 cm	188 (25.9%)	27 (27.8%)	17 (18.9%)	232 (25.4%)	
5–<10 cm	166 (22.8%)	27 (27.8%)	32 (35.6%)	225 (24.6%)	
≥10 cm	66 (9.1%)	17 (17.5%)	25 (27.8%)	108 (11.8%)	

BE, Barrett’s esophagus; C, Prague C length; LGD, low-grade dysplasia; M, Prague M length; ND, non-dysplastic; analysis done with Fisher’s exact test for categorical variables and ANOVA for continuous variables.

**Table 3:** Characteristics of patients with low-grade dysplasia

	LGD at entry (*n* = 97)	LGD during surveillance (*n* = 90)	All patients (*n* = 187)	*P*-value
**LGD variance**				0.151
Back and forth between LGD and ND BE	15 (15.5%)	22 (24.4%)	37 (19.8%)	
Reverted back to ND BE	68 (70.1%)	61 (67.8%)	129 (69.0%)	
Persistent LGD	14 (14.4%)	7 (7.8%)	21 (11.2%)	
**Change in maximum length of BE** ^*^				0.777
No change	83 (85.6%)	74 (82.2%)	157 (84.0%)	
Increase	7 (7.2%)	9 (10.0%)	16 (8.6%)	
Decrease	7 (7.2%)	7 (7.8%)	14 (7.5%)	
Only one confirmed biopsy of LGD	48 (49.5%)	49 (54.4%)	97 (51.9%)	0.498
Number of scopes before LGD diagnosis (median and range)	N/A	1 (1–9)	N/A	
Time in surveillance before LGD diagnosis (mean ± SD) (years)	N/A	3.6 ± 3.1	N/A	

BE, Barrett’s esophagus; LGD, low-grade dysplasia; ND, non-dysplastic.

^*^Length change was determined to be ≥2 cm change from index to the most recent endoscopy, analysis done with the chi-square test.

From the complete 914 patient cohort, 46 (5.0%) progressed to HGD/EAC. Within the subgroups, 25 (3.4%) progressed from the non-dysplastic BE group, 7 (7.2%) from the LGD at entry group, and 14 (15.6%) from the LGD during surveillance group (*P* < 0.0001, Fisher’s exact test). Of the 46 cases of HGD/EAC, 30 were HGD and 16 were EAC. In the non-dysplastic group, 10 were EAC and 15 were HGD. For the LGD at entry group, one developed EAC and six developed HGD, and in the LGD during surveillance group, five developed EAC and nine developed HGD.

**Fig. 4:**
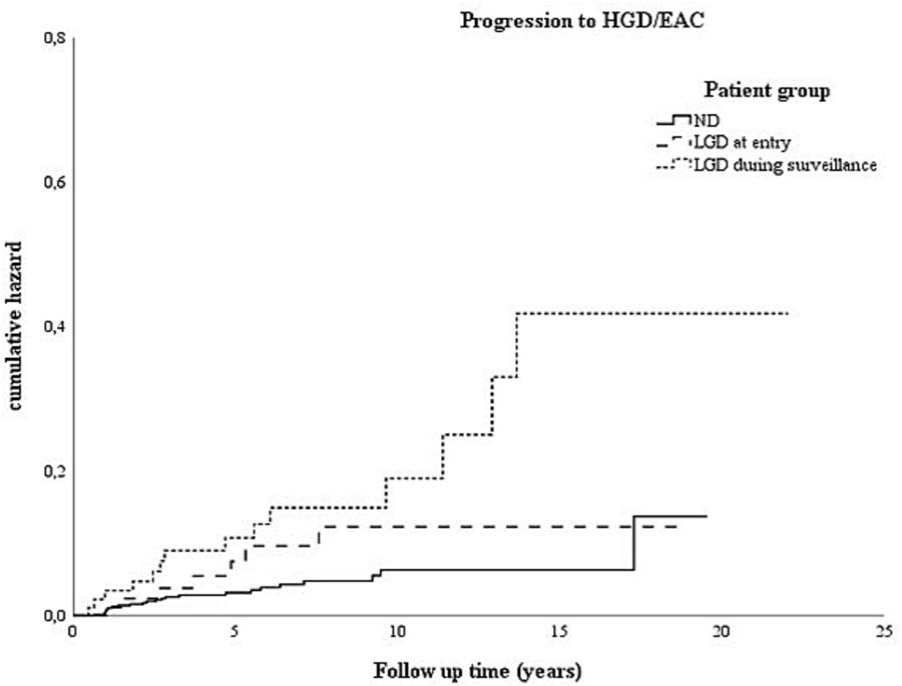
Kaplan–Meier chart of hazard/risk of progression to HGD/EAC. Log rank test *P* = 0.001.

The rate of progression to HGD/EAC per 100-person years was 0.6 (0.4–1.0) for the non-dysplastic BE group, 1.0 (0.4–2.1) for the LGD at entry group, and 2.2 (1.2–3.7) for the LGD during surveillance group. The multivariable Cox proportional hazard model is summarized in Tables 4 and 5 and showed a hazard ratio (HR) of 1.5 (0.6–3.5) for LGD at entry versus non-dysplastic BE and an HR of 2.4 (1.2–4.8) for LGD identified during surveillance versus non-dysplastic BE. Figure 4 depicts the Kaplan–Meier curve of cumulative hazard/risk of progression to HGD/EAC during the follow-up time for the respective groups.

**Table 4:** Rates of progression to high-grade dysplasia or esophageal adenocarcinoma

	ND (*n* = 727)	LGD at entry (*n* = 97)	LGD during surveillance (*n* = 90)	All patients (*n* = 914)	*P*-value
Crude rate of HGD/EAC	25 (3.4%)	7 (7.2%)	14 (15.6%)	46 (5.0%)	<0.001
Incidence rate of HGD/EAC (100- person year (95% CI))	0.6 (0.4–1.0)	1.0 (0.4–2.1)	2.2 (1.2–3.7)	0.9 (0.6–1.2)	*P* = 0.301/<0.001
IRR (95% CI)	1	1.6 (0.6–3.7)	3.4 (1.6–6.8)		*P* = 0.313/<0.001
HR (95% CI)	1	1.7 (0.7–3.9)	3.3 (1.7–6.4)		*P* = 0.236/<0.001

EAC, esophageal adenocarcinoma; HGD, high-grade dysplasia; HR, hazard ratio; IRR, incidence rate ratio; LGD, low-grade dysplasia; ND, non-dysplastic. Analysis for IRR and HR uses the ND group as a reference, and *P*-values are calculated with the chi-square test and Cox proportional hazard, respectively.

**Table 5:** Multivariable Cox proportional hazard for risk of progression to high-grade dysplasia or esophageal adenocarcinoma

	ND (ref)	LGD at entry HR (95% CI)	LGD during surveillance HR (95% CI)	*P*-value
Unadjusted	1	1.7 (0.7–3.9)	3.3 (1.7–6.4)	*P* = 0.236/<0.001
Adjusted for gender	1	1.9 (0.8–4.6)	3.7 (1.9–7.3)	*P* = 0.127/<0.001
Adjusted for length	1	1.6 (0.7–3.7)	2.6 (1.3–5.2)	*P* = 0.298/0.008
Adjusted for smoking	1	1.5 (0.6–3.6)	2.5 (1.2–5.0)	*P* = 0.331/0.010
Adjusted for age	1	1.5 (0.6–3.5)	2.4 (1.2–4.8)	*P* = 0.391/0.016

LGD, low-grade dysplasia; ND, non-dysplastic. Multivariable cox proportional hazard evaluating impact of LGD at entry or during surveillance compared to ND on risk of progressing to high-grade dysplasia or esophageal adenocarcinoma, adjusted for gender, index M length, smoking, and age. Each step includes the variable for the previous step. Last row is the final model including all above-mentioned variables.

### Cost-effectiveness of treatment strategies for LGD

A base case analysis was undertaken to determine the cost of managing an individual developing LGD. This base case strategy entailed an endoscopy 6 months after each LGD diagnosis with options to either progress to HGD/EAC and exit surveillance, continue with LGD, or revert to non-dysplastic BE (see Fig. 1). If patients reverted to non-dysplastic BE, then the surveillance interval lengthened to 12 months for the next endoscopy and then to 2 years if free from dysplasia. This strategy cost ~AU$46,884 per patient over a time horizon of 35 years and yielded 24.048 QALYs.

When RFA ablation was used to treat LGD, this was only cost-effective when applied to patients progressing from non-dysplastic BE at index endoscopy to LGD under surveillance, whereas continuing endoscopy surveillance was more cost-effective for individuals entering surveillance with a diagnosis of LGD at index endoscopy. Adding RFA to individuals progressing from non-dysplastic BE cost AU$53,511 (+$6626 more than the base strategy) and yielded 24.296 QALYs (+0.248 more than the base strategy), with an incremental cost-effective ratio (ICER) of AU$26,763. This was cost-effective at the willingness-to-pay threshold of AU$50,000/QALY. This was the only cost-effective ablation strategy. Performing RFA for patients entering the program with an index diagnosis of LGD was not cost-effective as it cost AU$62,091 (+$15,207) and yielded 23.84 QALY (−0.208), likely due to the disutility associated with procedures without a compensatory reduction in progression to EAC (see Table 6 and supplementary Figure 1–4).

**Table 6:** Health economic analysis for radiofrequency ablation for low-grade dysplasia

Strategy	Cost (AU$)	Change in cost	QALY	Change in QALY	ICER (AU$/QALY)	NMB (AU$)	Expected incidence of esophageal adenocarcinoma
Surveillance only for all LGD	$46,884 ± $9583		24.0 ± 0.1			$1,155,520 ± $10,928	8.8%
RFA for LGD at index endoscopy	$62,091 ± $6625	+$15,207	23.8 ± 0.1	−0.2	Inferior strategy	$1,129,929 ± $10,279	3.5%
RFA for LGD found during surveillance (ND at index endoscopy)	$53,511 ± $5316	+$6626	24.3 ± 0.1	+0.3	26,763	$1,161,273 ± $8248	8.3%
RFA for all LGD (index and surveillance endoscopy)	$68,718 ± $3904	+$21,834	24.1 ± 0.2	+0.1	547,031	$1,135,682 ± $11,973	3.1%

ICER, incremental cost-effectiveness ratio; LGD, low-grade dysplasia; ND, non-dysplastic; NMB, net monetary benefit; QALY, quality of adjusted life years; RFA, radiofrequency ablation.

Cost = mean cost in AUD$ ± standard deviation for each strategy. QALY = expected remaining quality-adjusted life years. ICER, incremental cost-effectiveness ratio = cost increase of a strategy divided by the QALY gained compared to the base strategy.

#### Deterministic sensitivity analysis

One-way sensitivity analysis, which varied one input value at a time, revealed the key drivers of the model in descending order were disutility associated with RFA, cost of diagnostic endoscopy, QALYs associated with cancer at stage I (amenable to EMR), cost of RFA, and transition probability of complications after RFA. When comparing the base-strategy (surveillance for LGD) versus the cost-effective strategy of RFA for LGD progressors on surveillance, only one variable—disutility associated with RFA (worse than 0.331), increased the ICER value above the willingness-to-pay threshold of AU$50,000/QALY. This reduction in utility, although temporary, is equivalent to having metastatic esophageal cancer and is thus deemed unrealistic for this scenario.

#### Probabilistic sensitivity analysis

Figure 2 depicts the incremental cost-effectiveness plane of 5000 simulations. Points below the willingness-to-pay threshold line are considered cost-effective (3424/5000 simulations of RFA for LGD progressors, 330/5000 for RFA for all LGD, and 3/5000 for RFA for LGD at the index endoscopy). Supplementary Figure 4 shows the cost-effectiveness acceptability curve, which shows the percent of simulations the endoscopic surveillance strategy was most cost-effective for different willingness to pay thresholds (0-AU$100,000/QALY). At low willingness-to-pay thresholds (<AU$25,000/QALY), LGD surveillance was cost-effective in >50% of the simulations. For willingness-to-pay thresholds >~AU$27,000/QALY, the RFA of the LGD progressors within the surveillance program was cost-effective in >50% of simulations. At a willingness-to-pay threshold of AU$50,000/QALY, the RFA of the LGD progressors in the surveillance program was cost-effective in ~78% of the simulations.

## DISCUSSION

The risk of progression to HGD/EAC following a pathological diagnosis of LGD varies widely in the published literature.^11^^,^^12^ While the majority of studies report progression rates of 1.5–2.0 per 100 person-years or similar, the much higher progression rate of 13.4 per 100 person-years per year reported by Phoa *et al*.^13^ from their randomized trial of RFA ablation versus surveillance for LGD in BE is an outlier, as their high progression rate has not been seen in other studies.^12^ These different rates are likely to reflect different criteria used to diagnose LGD in BE and different clinical contexts.

Our study, from a large tertiary referral center in South Australia, identified progression rates from LGD to HGD/EAC of 1.0 and 2.2 per 100 person-years. These findings are consistent with the 1.73 per 100 person-years progression rate reported by Singh *et al*. in their meta-analysis of LGD progression rates.^12^ However, unlike other studies, we separately determined the progression rates from LGD at entry into surveillance versus LGD identified during surveillance, with LGD identified during surveillance found to progress at a significantly higher rate. This might be explained by inconsistencies between pathologists diagnosing LGD in BE, the impact of active gastroesophageal reflux on the histopathological appearances leading to overdiagnosis of LGD, and histopathological assessment at the index endoscopy being conducted by a mix of expert and non-expert pathologists. The problem of inconsistencies with the diagnosis of LGD has been highlighted previously by Curvers *et al.*^25^

The different progression rates for the two LGD cohorts in our study outline the issues of overcalling LGD outside of surveillance programs. As many of the index endoscopies were performed for the assessment of gastroesophageal reflux symptoms, and BE was an incidental finding at that endoscopy, reflux control was not optimized before the index endoscopy for many of these patients. This contrasts with the patients under surveillance in whom significant efforts were made to ensure reflux symptom control using high-dose PPI therapy and occasionally anti-reflux surgery. Hence, in this cohort surveillance, endoscopy biopsies were likely easier to interpret, and those biopsies were also assessed by pathologists with an interest in gastrointestinal pathology. The difference in progression rates and the likely reasons for this highlight the need to be careful not to overinterpret a pathology report describing LGD in BE but rather to be aware of the context surrounding the collection of the biopsy specimens. In patients with LGD at the index endoscopy, we suggest that reflux control should be optimized, and then, endoscopy should be repeated to confirm that LGD is indeed present, before considering any intervention.

Despite the progression rate of 2.2 per 100 person-years from LGD at a surveillance endoscopy to HGD or mucosal cancer being well below the 13.4 per 100 person-years rate reported by Phoa *et al*.,^13^ this progression rate was still sufficiently high to justify ablation. However, it should be emphasized that the health economic analysis demonstrated cost-effectiveness for ablation using RFA only in patients developing LGD during surveillance and not in those diagnosed with LGD at the index endoscopy. This reinforces the need to confirm that LGD is indeed present if reported at the index endoscopy, and to withhold ablation until compliant with PPI treatment and confirmation at second endoscopy, which was seen to be cost-effective. In our study, the incremental cost-effective ratio (ICER) of AU$26,763 for RFA ablation in these patients was well below the current Australian willingness-to-pay threshold of AU$50,000/QALY. Of note, the willingness-to-pay threshold is higher in the United Kingdom at GBP30,000 (approx. AU$60,000) and even higher in the USA at US$100,000 (approx. AU$150,000). This suggests that the strategy of ablation once LGD is identified during surveillance will be cost-effective in other developed countries, provided their costs are similar to Australian health care system costs.

Conversely, ablation for LGD at the index endoscopy was not cost-effective, likely due to the lower rate of progression to HGD/EAC, which is a consequence of the potential overdiagnosis of LGD in these individuals. For these patients, optimization of gastroesophageal reflux control and then repeat endoscopy again demonstrating LGD, is likely required before considering ablation. Reversion to non-dysplastic BE upon re-endoscopy likely reflects individuals with either an incorrect initial diagnosis or those with features of inflammation rather than dysplasia, for whom ongoing endoscopy surveillance is the most appropriate management strategy in the authors’ setting. Alternatively, a double-expert Gastrointestinal pathologist review of LGD diagnoses in patients newly diagnosed with BE must be considered before ablation, although this was not well implemented in our study. It is also important to highlight that in the cohort of patients evaluated in our study, no intervention such as ablation was performed for LGD in any patients. The potential impact of ablation of BE in individuals identified with LGD is only estimated in the health economic model in our study.

A potential weakness of our study is that the diagnosis of LGD at histopathology was usually not confirmed by a second pathologist. This might have led to overdiagnosis of LGD, and within the entire group of patients with an LGD diagnosis, 51.9% had a diagnosis of LGD at only one time and then reverted to non-dysplastic BE. How many of these initial LGD diagnoses would possibly be downgraded from LGD to non-dysplastic if a second pathologist reviewed that the diagnosis is unclear? However, the practical reality of many surveillance programs, including ours, is that double confirmation of LGD is not feasible as pathology resources are limited, so our findings likely reflect usual clinical practice in many centers. Nevertheless, LGD, even though not double confirmed, was still associated with a significantly increased risk of progression. The multivariable analysis adjusted for known confounding variables (Table 5) still showed an HR for progression of 1.5 (95% CIs 0.6–3.5) for LGD at entry and 2.4 (1.2–4.8) for LGD identified during surveillance compared to non-dysplastic BE.

Since in our study, we differentiated patients with LGD into either LGD at entry into surveillance vs. LGD arising during the surveillance, there might be potential for lead time/immortal time bias. The time to progression in the LGD arising during the surveillance group was calculated from the time that the LGD was first identified and not the time of entry into the surveillance program. We chose to do so as this study focused on the risk of progression when a diagnosis of LGD is made. If we included the time under surveillance before the LGD diagnosis, then the follow-up time until progression to HGD/EAC would be longer, and the IRR and HR would be lower. However, that scenario is not applicable to the clinical setting.

A strength of our current study is that it analyzed prospectively collected surveillance data from a large cohort of 914 patients managed for more than two decades. The quality of the endoscopy procedures and biopsy collection was monitored across this period to ensure protocol compliance, and consistently high levels of compliance have been demonstrated in this cohort previously.^19^^,^^20^ Our finding of an annual progression rate of 2.2 per 100 person-years following identification of LGD in patients under surveillance is more likely to reflect the real world that most clinicians work in, rather than the 13.4 per 100 person-years progression rate reported in the context of Phoa *et al.*’s randomized trial.^13^ We suggest that this lower progression rate is more realistic and can be used to inform management strategies.

In conclusion, we have shown that the risk of progression to HGD/EAC in individuals diagnosed with LGD in BE is dependent on the clinical circumstances at the time of the LGD diagnosis. If identified at the index endoscopy, then the risk of progression appears to be too low and it is not cost-effective to intervene with RFA ablation. Optimizing reflux control and then repeating the endoscopy is likely the most appropriate next step in these individuals. However, we have also shown that LGD identified in patients under surveillance in a managed program is associated with a significantly higher risk of progression, and in these patients, RFA ablation of the BE segment is likely the most cost-effective management strategy for their LGD.

## Supplementary Material

final_version_supplementary_material_dis_esophagus_LGD_in_barrett_doag028

## Notes

Acknowledgments, funding, and competing interests: CHK received a research stipend from the Swedish Surgical Society and the Swedish Society of Medicine. RV was funded by a PhD grant from The Hospital Research Foundation (SAPhD000032018). DW received a Beat Cancer Grant from the Cancer Council of South Australia, supported by the Department of Health and the Flinders Medical Centre Foundation, and this funded NB’s salary. All other authors have nothing to declare. The funders had no impact on the conceptualization, analysis, interpretation, drafting, and decision to publish.

Specific author contributions: CHK, RV, AS, TB, NB, and DW planned and conceptualized the study. AS, CHK, and RV collected data and conducted data analysis. All authors were involved in data analysis. CHK and RV drafted the manuscript. CHK and RV contributed equally to the study. All authors were involved in manuscript revision for intellectual content and approval of final draft.

Financial support: None.

Potential competing interests: None.

Financial disclosure: The authors have no financial disclosures to make. There were no conflicts of interest.
