# Supplementary material for: Impact of a diagnosis of ‘low-grade dysplasia’ in patients with Barrett’s esophagus
Source: Dis Esophagus. 2026 Apr 9;39(2):doag028. doi: 10.1093/dote/doag028 (PMC13070377; doi:10.1093/dote/doag028)
Supplement: final_version_supplementary_material_dis_esophagus_LGD_in_barrett_doag028 [file final_version_supplementary_material_dis_esophagus_lgd_in_barrett_doag028.docx]

**Supplementary Material**

**Supplementary Table 1.**

| Risk-related health states | LGD at entry | LGD from surv |
| --- | --- | --- |
| No Barrett's to NDBE | 21.5% | 21.5% |
| No Barrett's to LGD | 0.2% | 0.2% |
| No Barrett's to HGD | 0.03% | 0.03% |
| NDBE to No Barrett’s | 17.2% | 17.2% |
| NDBE to LGD | 4.2% | 4.2% |
| NDBE to HGD | 0.1% | 0.1% |
| NDBE to EAC | 0.1% | 0.1% |
| LGD to HGD | 1.2% | 1.3% |
| LGD to EAC | 0.1% | 0.7% |
| LGD to NDBE | 19.5% | 9.6% |

**Supplementary Table 2. Separate reference list for supplemental material**

| **Description** | **Mean value** | **Standard deviation** | **Ref** |
| --- | --- | --- | --- |
| Probability of complication post endoscopic treatment (RFA or EMR) | 20% | 14% | [1] |
| Probability of requiring re-treatment with RFA and/or EMR (HGD and EAC) | 8.8% | 11.9% | [2, 3] |
| Probability of HGD progression to adenocarcinoma post endoscopic treatment | 1% | 0.6% | [3, 4] |
| Probability of LGD progression to HGD post endoscopic treatment | 0.5% | 0.1% | [2, 4, 5] |
| Probability of requiring re-ablation of LGD lesion | 9.7% | 0.8% | [6-8] |
| Mortality post endoscopic treatment | 0.6% | 0.1% | [5] |
| Localised cancer | 19% | - | [9] |
| Regional spread | 29% | - | [9] |
| Unstaged cancer | 20% | - | [9] |
| Distant spread | 32% | - | [9] |
| Background mortality (age dependent) | Life-Tables |  | [10] |

RFA- Radiofrequency ablation; EMR- endoscopic mucosal resection; HGD- high grade dysplasia; EAC- esophageal adenocarcinoma; LGD- low grade dysplasia

**Supplementary Table 3. Separate reference list for supplemental material**

|  | **Cost in AU$ (std dev)** | **Ref** |
| --- | --- | --- |
| Cost of an endoscopy | $1,400 (± $400) | SA database |
| Cost of maintaining database (per individual annually) | $185 | [11] Gordon 2014 (CPI adjusted) |
| Cost of radiofrequency ablation | $10,000 (± $2,490) | SA database |
| Cost of endoscopic mucosal resection | $14,000 (± $4,000) | SA database |
| Cost of complication post endoscopic intervention | $12,300 (± $3,600) | SA database |
| Cost of curative treatment (trimodality) | $95,450 (± $37,000) | [11, 12] |
| Cost of palliation | $15,700 (± $2,412) | [11, 12] |
|  |  |  |
|  | **QALYs** | **Ref** |
| Background utility | Age Dependent | [11, 13] Viney 30 |
| Surveillance detected cancer | Background utility - 0.1 (± 0.01) | [11, 14] Sullivan 34 |
| Localised Cancer (no nodal spread) | Background utility - 0.2 (± 0.02) | [11, 15] Garside 32, Gerson 33 |
| Regional Spread | Background utility - 0.2 (± 0.03) | [11, 14, 15] de boer 31 |
| Metastatic cancer | Background utility - 0.3 (± 0.04) | [11, 14, 15] de boer 31 |
| Unstaged Cancer | Background utility - 0.2 (± 0.01) | [11] |
| ***Disutility*** for complication post endoscopic intervention (1 cycle) | -0.05 (± 0.01) | [11] |
| ***Disutility*** associated with endoscopic intervention (1 cycle) | -0.04 (± 0.01) | [11] |

Trimodality- Neoadjuvant chemoradiotherapy followed by surgical resection; Endoscopic mucosal resection- includes cost of radiofrequency ablation

**Supplementary Figure 1**


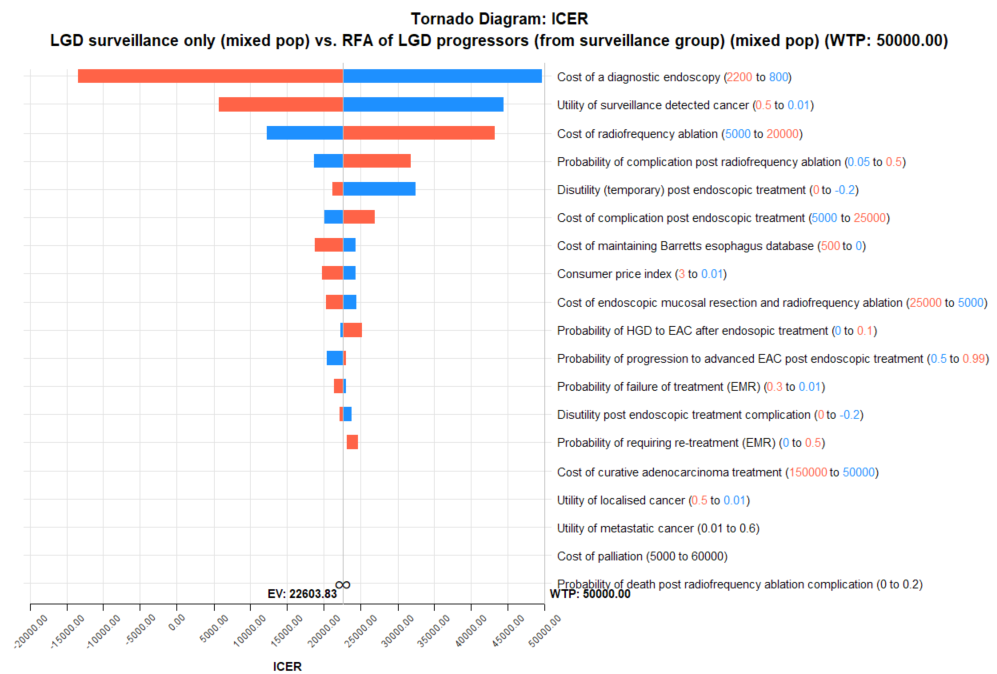


**Supplementary Figure 2**
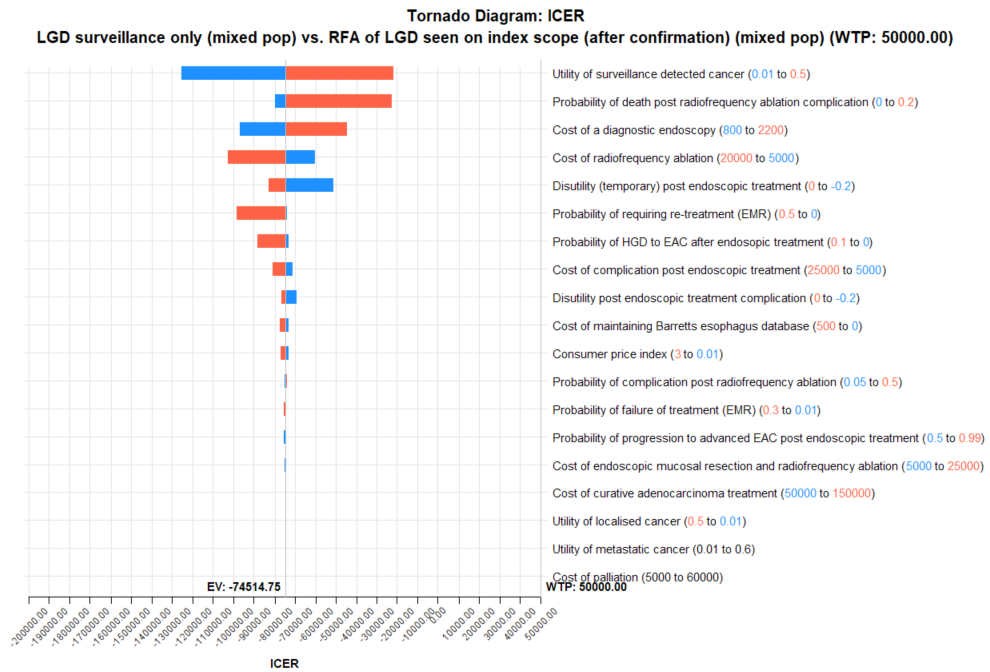


**Supplementary Figure 3**


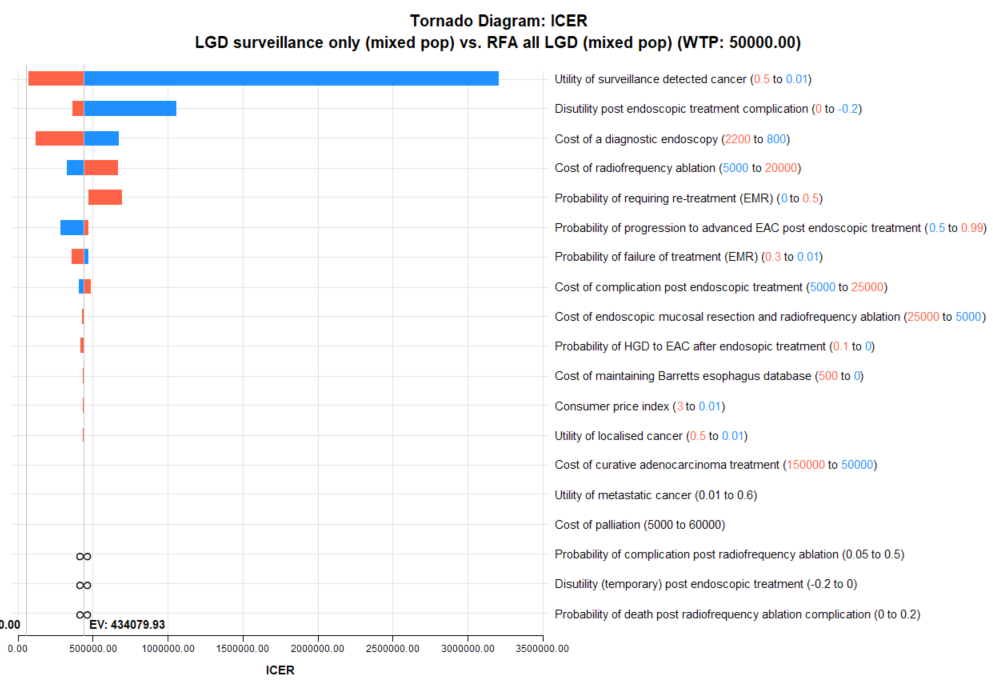


**Supplementary Figure 4**


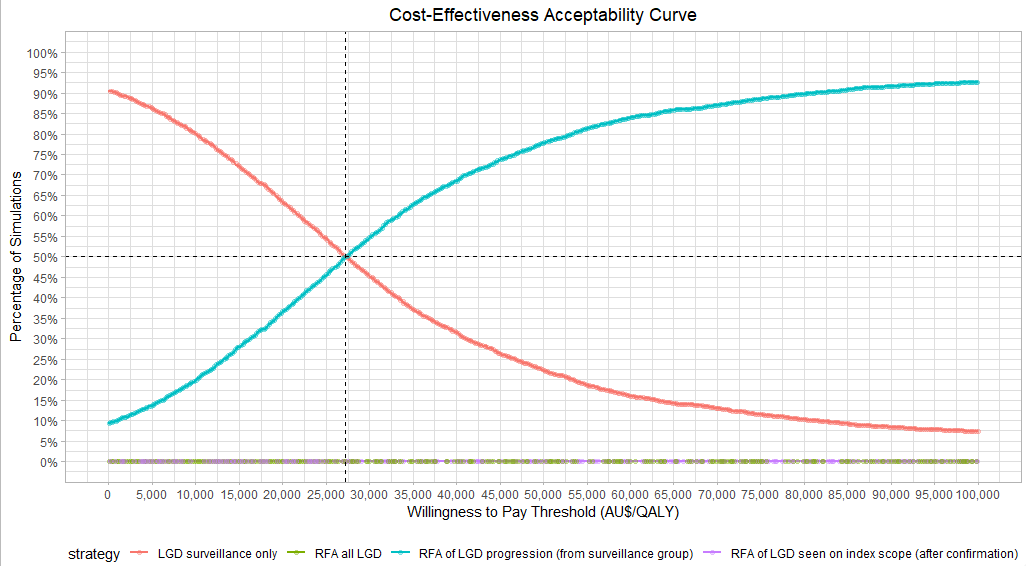
**Supplementary material reference list**

1. Tomizawa, Y., et al., *Efficacy, Durability, and Safety of Complete Endoscopic Mucosal Resection of Barrett Esophagus: A Systematic Review and Meta-Analysis.* J Clin Gastroenterol, 2018. **52**(3): p. 210-216.

2. Phoa, K.N., et al., *Remission of Barrett's esophagus with early neoplasia 5 years after radiofrequency ablation with endoscopic resection: a Netherlands cohort study.* Gastroenterology, 2013. **145**(1): p. 96-104.

3. Pouw, R.E., et al., *Efficacy of radiofrequency ablation combined with endoscopic resection for barrett's esophagus with early neoplasia.* Clin Gastroenterol Hepatol, 2010. **8**(1): p. 23-9.

4. Wolf, W.A., et al., *Incidence of Esophageal Adenocarcinoma and Causes of Mortality After Radiofrequency Ablation of Barrett's Esophagus.* Gastroenterology, 2015. **149**(7): p. 1752-1761 e1.

5. Qumseya, B.J., et al., *Disease Progression in Barrett's Low-Grade Dysplasia With Radiofrequency Ablation Compared With Surveillance: Systematic Review and Meta-Analysis.* Am J Gastroenterol, 2017. **112**(6): p. 849-865.

6. Small, A.J., et al., *Radiofrequency Ablation Is Associated With Decreased Neoplastic Progression in Patients With Barrett's Esophagus and Confirmed Low-Grade Dysplasia.* Gastroenterology, 2015. **149**(3): p. 567-76 e3; quiz e13-4.

7. Guthikonda, A., et al., *Clinical Outcomes Following Recurrence of Intestinal Metaplasia After Successful Treatment of Barrett's Esophagus With Radiofrequency Ablation.* Am J Gastroenterol, 2017. **112**(1): p. 87-94.

8. Pandey, G., et al., *Systematic review and meta-analysis of the effectiveness of radiofrequency ablation in low grade dysplastic Barrett's esophagus.* Endoscopy, 2018. **50**(10): p. 953-960.

9. SEER-Database, *Surveillance, Epidemiology, and End Results Program Cancer Stat Facts: Esophageal Cancer*. 2020, National Cancer Institute: Bethesda, Maryland.

10. Australian-Bureau-of-Statistics, *Life tables 2017-2019*, in *2020*. 2020.

11. Gordon, L.G., et al., *Cost-effectiveness of endoscopic surveillance of non-dysplastic Barrett's esophagus.* Gastrointestinal Endoscopy, 2014. **79**(2): p. 242-256.e6.

12. Gordon, L.G., et al., *Modeling the cost-effectiveness of strategies for treating esophageal adenocarcinoma and high-grade dysplasia.* J Gastrointest Surg, 2012. **16**(8): p. 1451-61.

13. Viney, R., et al., *Time Trade-Off Derived EQ-5D Weights for Australia.* Value in Health, 2011. **14**(6): p. 928-936.

14. Wildi, S.M., et al., *Assessment of health state utilities and quality of life in patients with malignant esophageal Dysphagia.* Am J Gastroenterol, 2004. **99**(6): p. 1044-9.

15. McNamee, P., et al., *Chained time trade-off and standard gamble methods. Applications in oesophageal cancer.* Eur J Health Econ, 2004. **5**(1): p. 81-6.
